# Eosinophilic Granulomatosis With Polyangiitis (EGPA) Manifesting as Giant Cell Arteritis After Initiating Treatment With Dupilumab: An Unusual Clinical Presentation

**DOI:** 10.7759/cureus.106985

**Published:** 2026-04-13

**Authors:** Sameen Zafar, Enrique Medina, Roopkiran Panesar, Vijaya L Murthy

**Affiliations:** 1 Rheumatology, Baylor College of Medicine, Houston, USA; 2 Internal Medicine, University of Texas Medical Branch, Galveston, USA; 3 Rheumatology, University of Texas Medical Branch, Galveston, USA

**Keywords:** antineutrophil cytoplasmic antibody (anca)-associated vasculitis (aav), churg-strauss syndrome (css), dupilumab, eosinophilic granulomatosis with polyangiitis (egpa), giant cell arteritis (gca)

## Abstract

Eosinophilic granulomatosis with polyangiitis (EGPA) is a rare antineutrophil cytoplasmic antibody (ANCA)-associated multisystemic small vessel inflammatory disease. Its diverse clinical presentation overlaps both within its own phases and with other disorders, complicating diagnosis. We present a case of a 60-year-old female who developed symptoms suggestive of giant cell arteritis (GCA) with polymyalgia rheumatica (PMR) shortly after starting treatment with dupilumab for severe rhinosinusitis. On further evaluation, the temporal artery biopsy was negative for arteritis, and the skin biopsy confirmed the diagnosis of EGPA. This case emphasizes the importance of recognizing shared symptoms and constantly reassessing vasculitis patients. Timely and accurate diagnosis is crucial for unique treatment strategies tailored for each vasculitis. This case also brings attention to the fact that some anti-asthma drugs, including dupilumab, can potentially unmask pre-existing subclinical EGPA. Although there are few documented cases of EGPA diagnosed after dupilumab treatment, this association requires further investigation, as current literature is uncertain whether it acts as a trigger versus lacking a direct impact on disease treatment.

## Introduction

Eosinophilic granulomatosis with polyangiitis (EGPA), formerly known as Churg-Strauss syndrome, is an antineutrophil cytoplasmic antibody (ANCA)-associated multisystemic inflammatory disorder. EGPA presents with a highly variable clinical manifestation, as its phases may overlap and mimic other eosinophilic and vasculitic disorders. This complexity makes EGPA exceedingly difficult and challenging, resulting in delayed or missed diagnoses [1].

The 2022 American College of Rheumatology/European Alliance of Associations for Rheumatology (ACR/EULAR) Classification Criteria for Eosinophilic Granulomatosis with Polyangiitis was developed to aid in the diagnosis of EGPA among patients with suspected vasculitis. Positive scoring criteria include eosinophilia, obstructive airway disease, nasal polyps, extravascular eosinophilic inflammation, and mononeuritis multiplex, while negative scores are assigned for cytoplasmic ANCA (C-ANCA) pattern on immunofluorescence or anti-proteinase 3 (PR3)-ANCA positivity and hematuria [2].

An emerging clinical concern is the potential association between dupilumab therapy and the development or unmasking of EGPA. Dupilumab, a monoclonal antibody targeting the interleukin-4 receptor alpha subunit, is widely used in treating type 2 inflammatory conditions, including severe asthma and chronic rhinosinusitis with nasal polyps [3].

Although dupilumab is generally well tolerated, eosinophilic complications have been increasingly reported. A 2025 systematic review identified 53 reported cases of eosinophilic complications during dupilumab therapy globally, with EGPA being the most common (24 patients), typically developing after a median of nine weeks [4]. Additionally, rising peripheral eosinophil counts early after dupilumab initiation have been associated with an increased risk of subsequent EGPA diagnosis [5]. Despite these observations, such events remain exceedingly rare, and it remains unclear whether dupilumab directly induces vasculitis, unmasks subclinical disease, or alters disease expression through corticosteroid-sparing effects.

## Case presentation

This report discusses a 60-year-old Caucasian woman, a former smoker with a medical history significant for severe asthma, chronic allergic rhinosinusitis with nasal polyps, and peripheral neuropathy. She was initially treated with benralizumab for sinus disease but was transitioned to dupilumab due to inadequate response. Shortly thereafter, she developed worsening arthralgias, myalgias, and neuropathic symptoms. Neurologic evaluation revealed bilateral lower extremity peripheral sensory neuropathy, with examination findings consistent with involvement of both small and large sensory nerve fibers.

**Figure 1:**
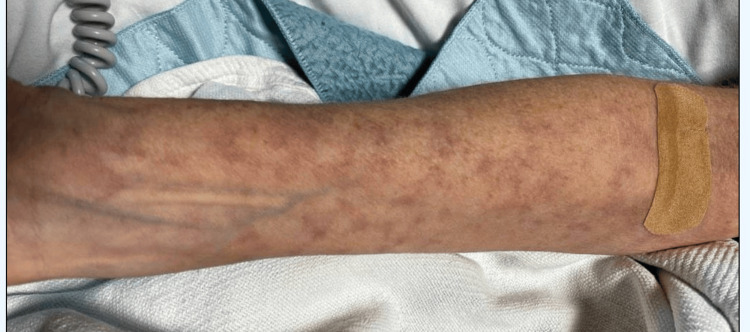
Nonpalpable livedo-racemose-like violaceous macules on the extensor surfaces of the upper extremities.

Approximately six weeks after initiating dupilumab and following her third dose, the patient presented with severe headaches, photophobia, jaw pain, and transient vision loss progressing to complete blindness, prompting evaluation in the emergency department. Additional symptoms included chills, nausea, hand weakness, and a violaceous rash on the upper extremities (Figure 1).

Initial laboratory evaluation demonstrated marked leukocytosis, eosinophilia, and elevated inflammatory markers. Further lab evaluation revealed positive anti-nuclear antibodies (ANA), anti-double-stranded DNA, and myeloperoxidase (MPO) ANCA (Table 1), with other autoimmunes including PR3 antibodies, anti-ribonucleoprotein, anti-Smith, thyroid peroxidase (TPO) antibodies, anti-SSA (RO), and anti-SSB (LA), and infectious studies including human immunodeficiency virus, syphilis, hepatitis, and QuantiFERON-TB Gold returning negative. Although ANA and anti-double-stranded DNA (dsDNA) were positive, there were no clinical features to suggest systemic lupus erythematosus or another connective tissue disease.

**Table 1:** Summary of laboratory findings during hospital evaluation All laboratory values were obtained during initial hospital evaluation. Abnormal results are categorized as high or abnormal. Reference ranges are provided for clarity.

Laboratory Parameter	Result	Reference Range	Interpretation
White blood cell count	21.82 ×10³/µL	4.30–11.10 ×10³/µL	High
Absolute eosinophil count	3.26 ×10³/µL	0.03–0.39 ×10³/µL	High
Erythrocyte sedimentation rate	112 mm/hr	2–30 mm/hr	High
Myeloperoxidase (MPO)-antineutrophil cytoplasmic antibody (ANCA)	34 U/mL	≤3.5 U/mL	High
C-reactive protein	17.9 mg/dL	<0.8 mg/dL	High
Anti–double-stranded DNA (anti-dsDNA)	5.0 IU/mL	0.0–4.0 IU/mL	High
Antinuclear antibody (ANA)	Positive (titer 1:80)	Negative	Abnormal

**Figure 2:**
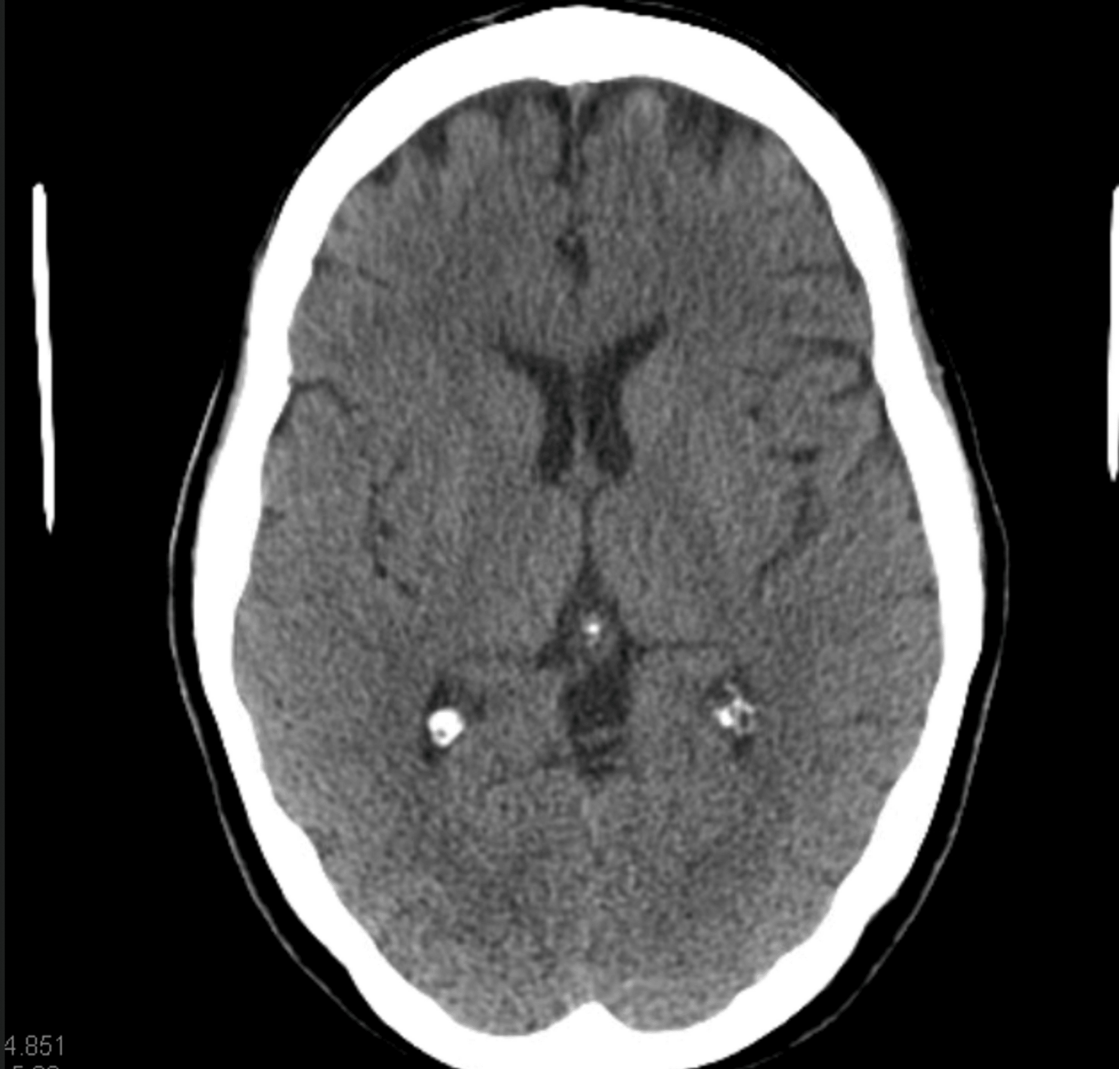
CT of the head without contrast showing no acute intracranial abnormality.

**Figure 3:**
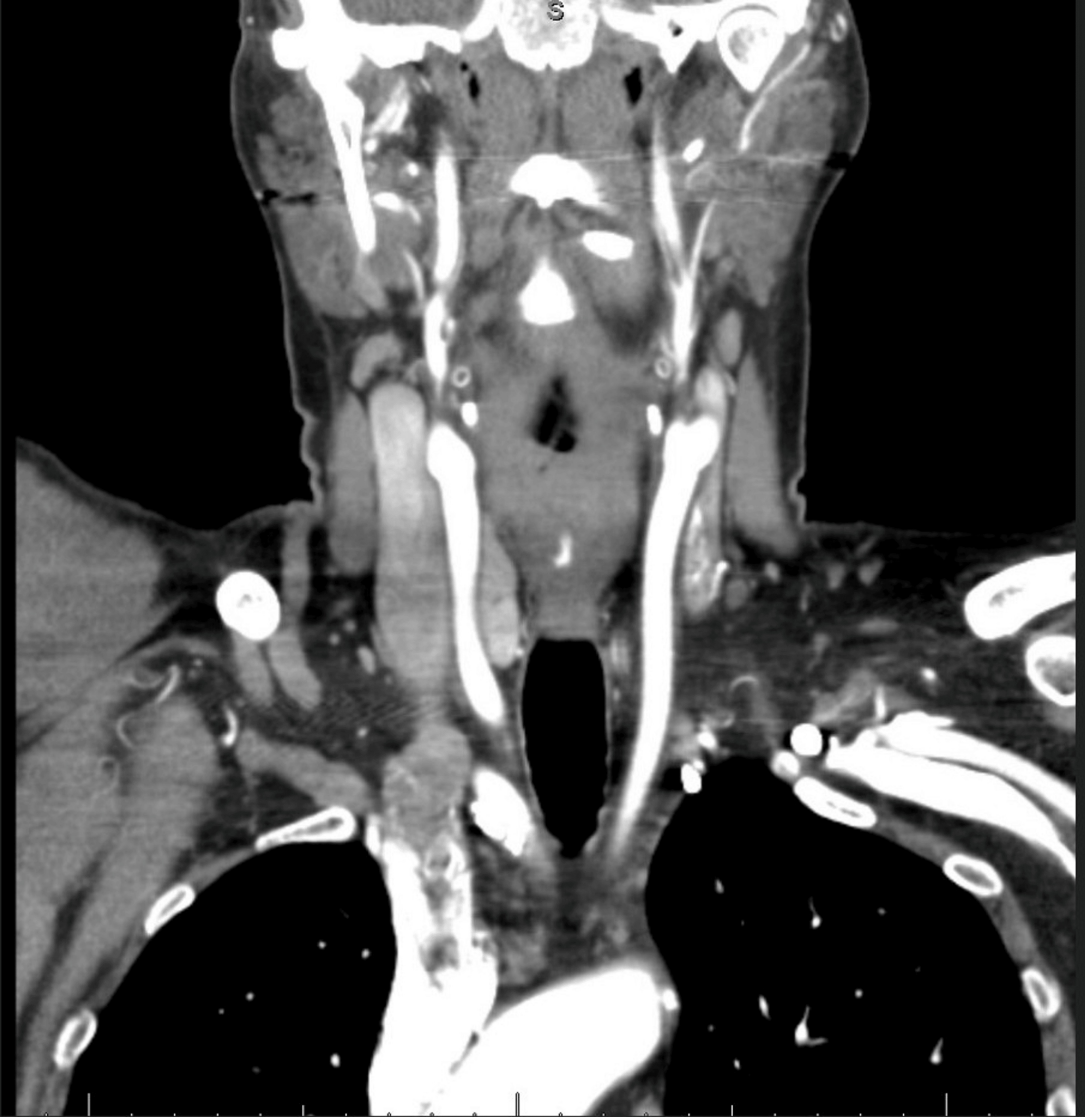
CT angiography of the head and neck demonstrating no evidence of vascular stenosis or aneurysm.

**Figure 4:**
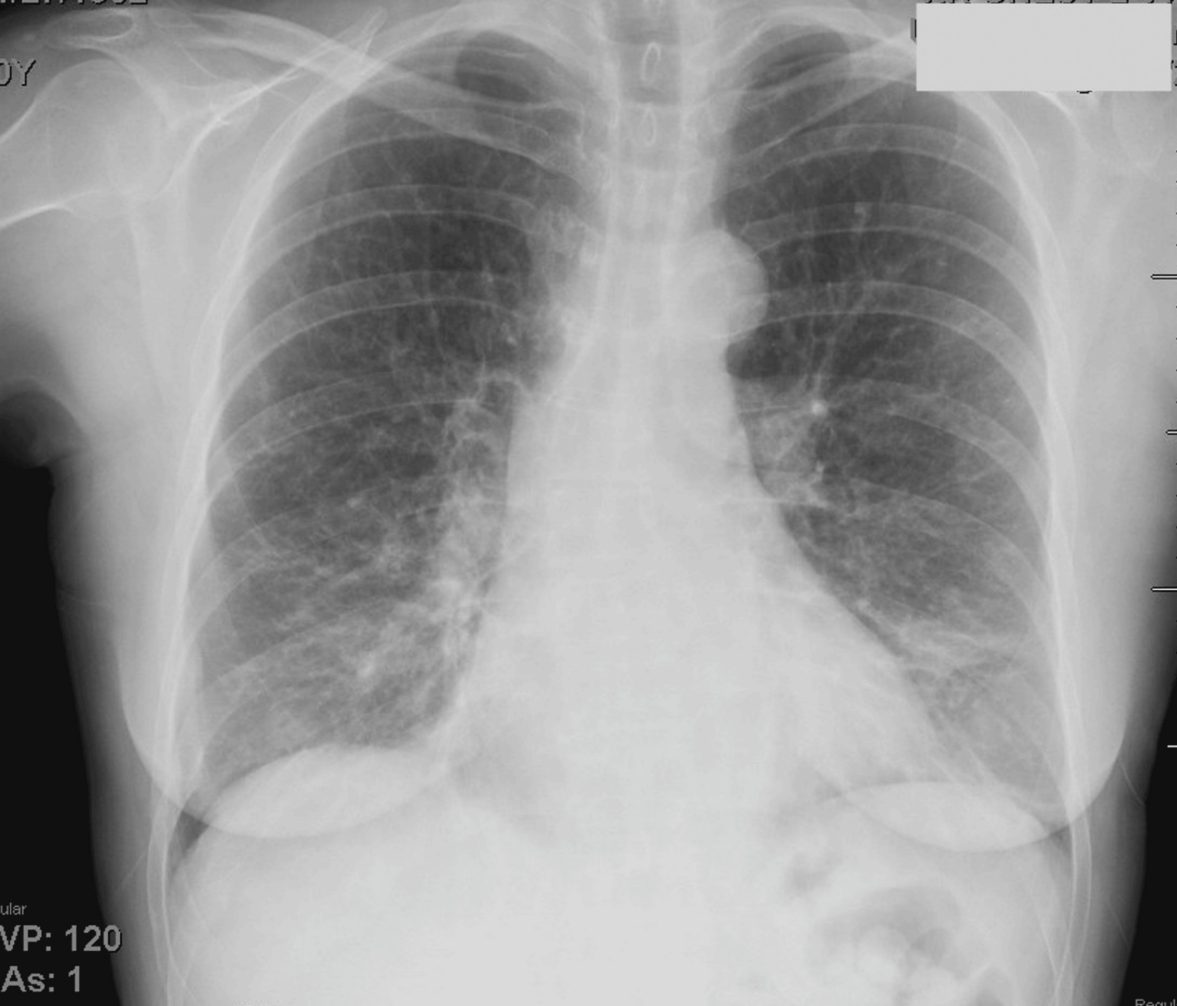
Chest radiograph demonstrating bilateral lower lobe opacities.

Neuroimaging (Figure 2) and vascular imaging (Figure 3) were unremarkable, while chest radiography (Figure 4) revealed lower lobe opacities.

**Figure 5:**
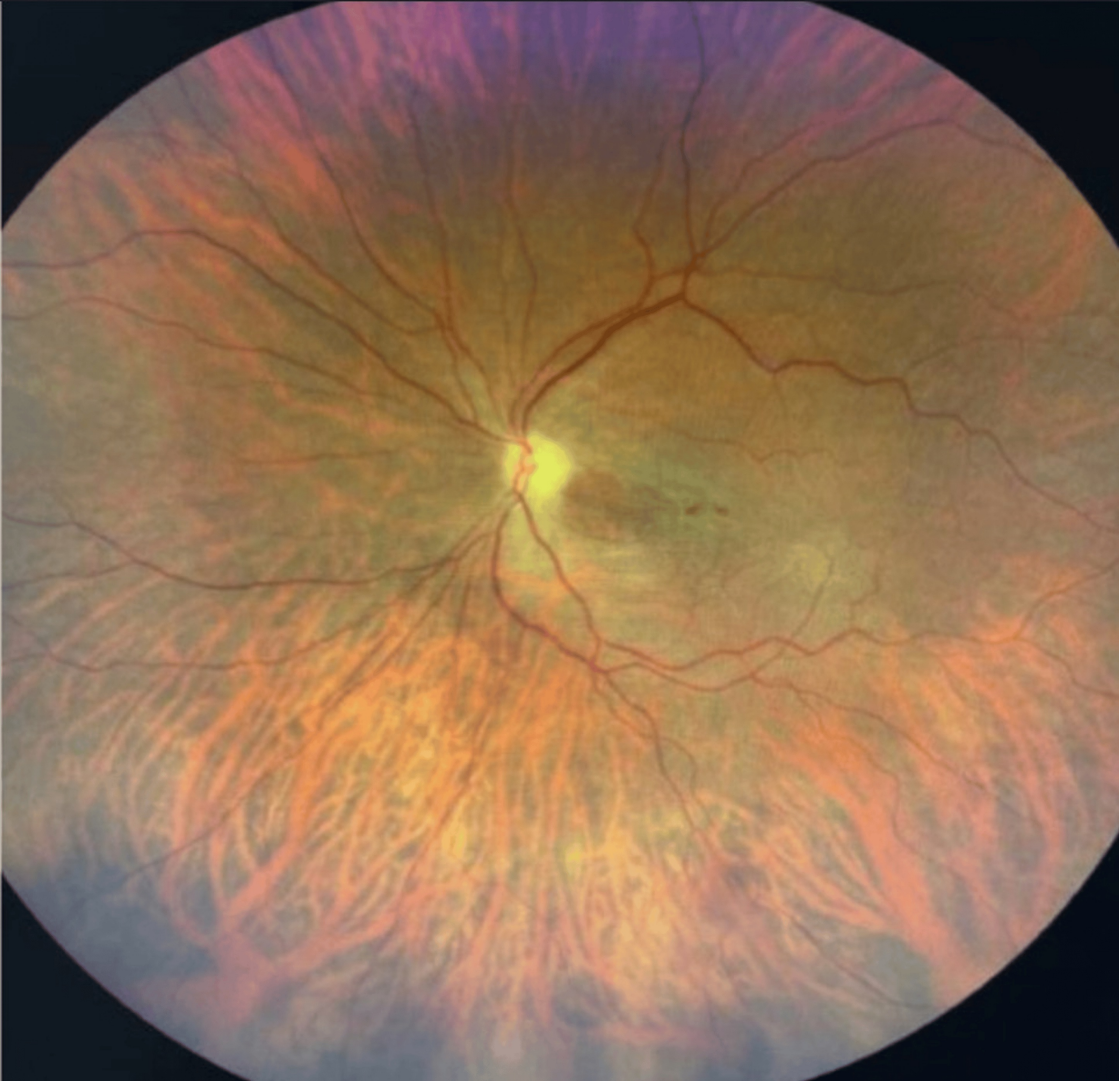
Ophthalmology exam showing central retinal artery occlusion.

Ophthalmologic evaluation confirmed central retinal artery occlusion (Figure 5).

Given severe temporal headache and jaw claudication concerning for giant cell arteritis (GCA) with polymyalgia rheumatica (PMR), she was started on IV methylprednisolone.

**Figure 6:**
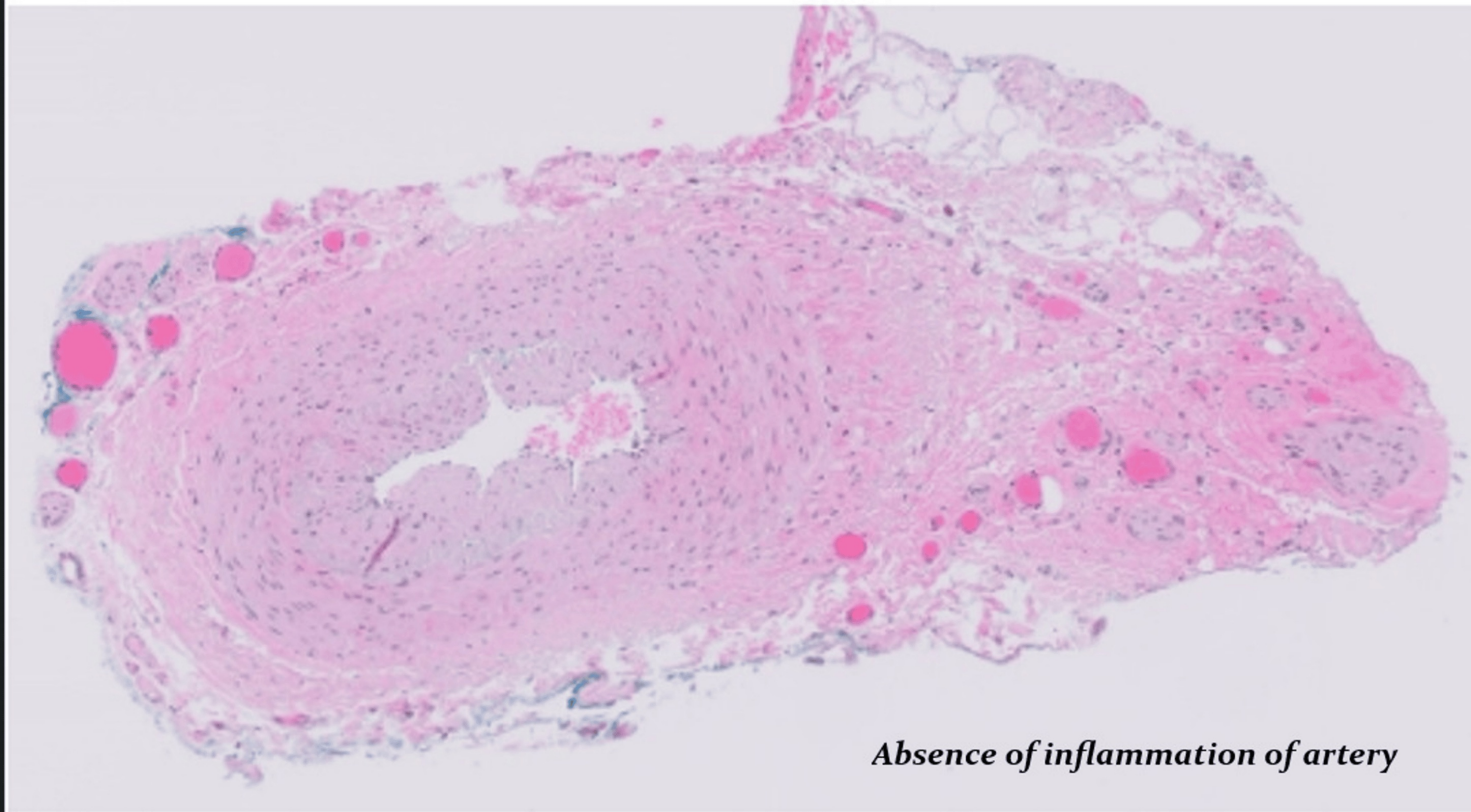
Section of the temporal artery stained with the routine H&E; there are no signs of inflammation of the artery.

**Figure 7:**
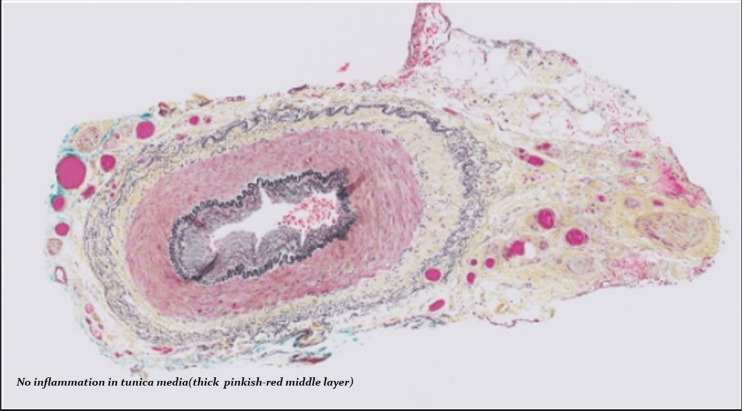
Sequential section of the temporal artery negative for arteritis, stained with the Movat stain. The Movat stain reveals an intact internal elastic lamina (dark, wavy ring) and the absence of fibrosis in the tunica media (pinkish-red middle layer) without any inflammation.

Echocardiogram with bubble study showed an extracardiac shunt, and further evaluation was decided to be done as an outpatient procedure by cardiology. Temporal artery biopsy (Figure 6 for H&E staining of biopsy and Figure 7 for Movat staining of biopsy) performed by vascular surgery three days after initiation of corticosteroid therapy was negative for arteritis.

**Figure 8:**
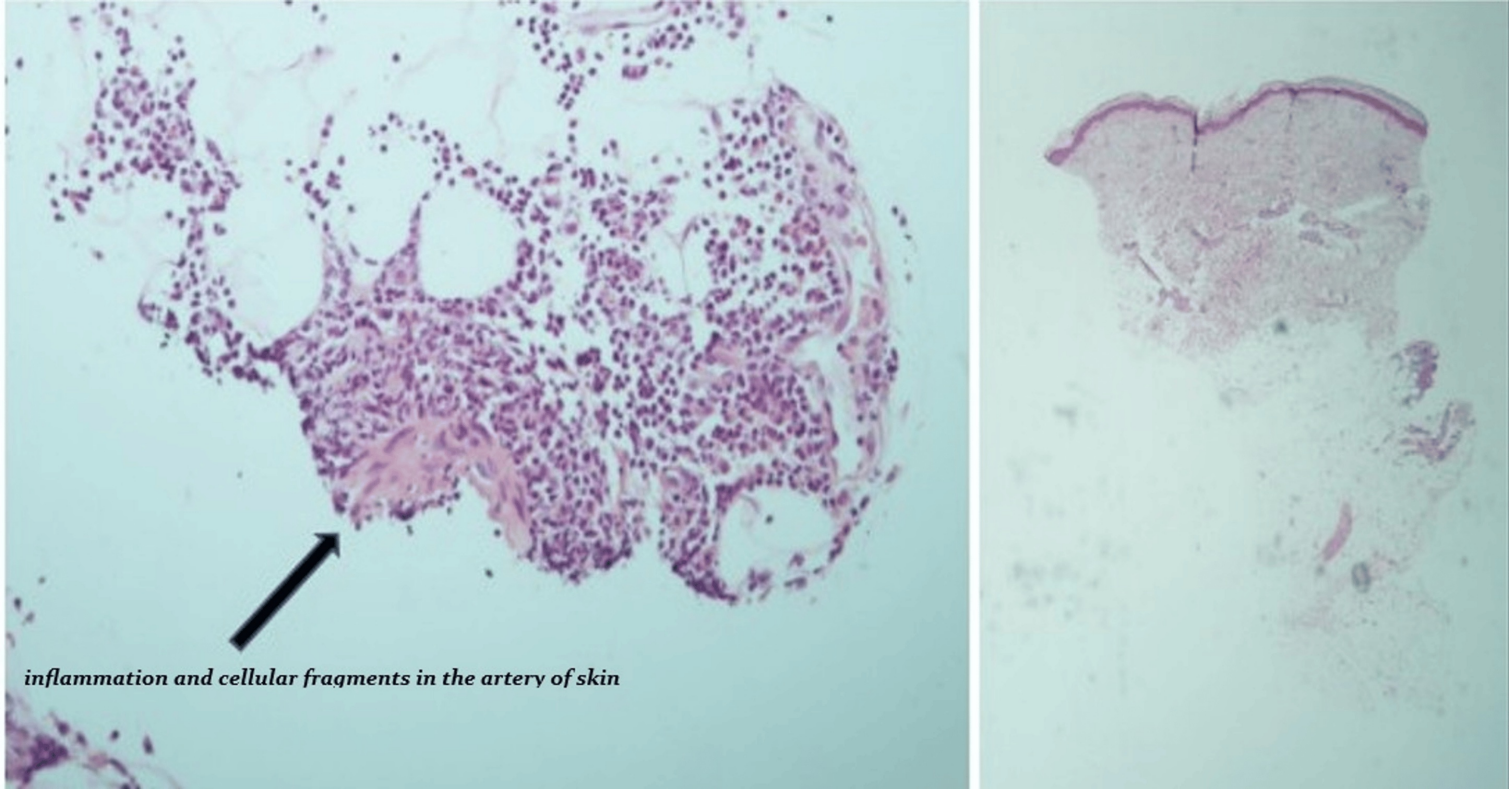
Skin biopsy from the right superior ventral forearm showing leukocytoclastic and eosinophilic vasculitis of a medium-sized subcutaneous artery. The black arrow points to the area of vascular inflammation and cellular fragments in the skin biopsy.

Given her presentation with severe asthma, chronic sinusitis, nasal polyps, eosinophilia, pulmonary infiltrates, livedo-like skin lesions, and polyneuropathy, EGPA was suspected. Skin biopsy was done, demonstrating leukocytoclastic vasculitis with prominent eosinophilic infiltration and extravascular eosinophils in a medium-sized subcutaneous artery (Figure 8), establishing the diagnosis. She met the ACR/EULAR 2022 classification criteria for EGPA with a total score of 13 (obstructive airway disease +3, nasal polyps +3, elevated blood eosinophil count +5, and biopsy supporting vasculitis +2) [2]. Rituximab therapy was initiated, and the patient was discharged with close rheumatology and allergy/immunology follow-up.

## Discussion

Here, through this report, we are discussing the unique case of a 60-year-old female patient initially suspected of having GCA with PMR based on vision loss, jaw claudication, severe temporal headache, and arthralgias, who was subsequently diagnosed with EGPA. This presentation is considered atypical and extremely rare. Two recent case reports highlighted temporal arteritis as an initial clinical manifestation of EGPA [6,7]. In one of them, Nishimura et al. reviewed 10 similar cases demonstrating temporal artery involvement associated with the EGPA [7]. Unlike previously reported cases, our case is unique as initially, symptoms mimicked GCA with PMR clinically, but the temporal artery biopsy was negative for arteritis. Although the sensitivity of temporal artery biopsy can vary widely, ranging from 70% to 90%, with lower positivity rates (ranging 15% to 40%) in certain clinical settings, CA cannot be entirely excluded based on biopsy results alone. However, the patient’s overall clinical presentation, laboratory findings, and biopsy results from other sites collectively supported a diagnosis of EGPA after further evaluation. While glucocorticoids are administered as initial treatment for both conditions, differentiating between GCA and EGPA is crucial for tailored treatment strategies, involving the addition of cyclophosphamide or rituximab for EGPA and tocilizumab [1], upadacitinib [8], or methotrexate for GCA [9].

This case indicates the importance of recognizing shared symptoms and consistently reassessing vasculitis patients to avoid clinical biases such as diagnostic momentum and anchoring.

Another notable aspect of this case is the patient’s prior biologic therapy. She had initially received benralizumab for two months, which was switched to dupilumab (Dupixent) due to inadequate response. Within two weeks of starting Dupixent, she developed generalized arthralgias and intermittent numbness/neuropathy, highlighting the need to consider the rare association of dupilumab with EGPA manifestations.

A review of the literature identifies few cases of EGPA diagnosed following dupilumab therapy in asthma patients. Tanaka et al. reported a 50-year-old male patient diagnosed with EGPA five months after initiating treatment with dupilumab, without concurrent oral corticosteroids or other monoclonal antibodies [10]. Persaud et al. also described an association of EGPA manifestations shortly after dupilumab initiation [11]. Similarly, Yamazaki et al. highlighted another case of a 77-year-old female patient on oral prednisolone who developed EGPA symptoms six months after starting dupilumab [12]. Additional reports have discussed dupilumab triggering vasculitis in MPO-ANCA-positive patients [13].

Conversely, Ikeda et al. reported a case in which EGPA became apparent a few months after discontinuing dupilumab, raising concerns that EGPA symptoms might be masked during dupilumab use [14].

In our case, while we noticed an association in the clinical manifestation of EGPA after initiating dupilumab, it remains unclear whether it acted as a trigger for the development of the vasculitis phase of the disease.

Several mechanisms, including immune modulation and unmasking of underlying eosinophilic disease, have been proposed to explain a potential link between dupilumab and EGPA; however, these remain speculative and observational. Our findings highlight the need for further evaluation to clarify the potential relationship between dupilumab and EGPA.

## Conclusions

This case underscores the diagnostic complexity of EGPA, particularly when its clinical features overlap with other vasculitides such as GCA. It highlights the importance of maintaining a broad differential diagnosis, continually reassessing clinical assumptions, and integrating laboratory and histopathologic findings to achieve an accurate diagnosis. Recognition of EGPA is critical, as treatment strategies differ substantially from those of other inflammatory vasculitides. The temporal association with dupilumab therapy raises awareness of a potential link between biologic treatment and the emergence or unmasking of EGPA, emphasizing the need for clinical vigilance when new or worsening eosinophilic or vasculitic symptoms appear, even in the absence of classic presentations. Ultimately, this case reinforces the value of personalized, timely management and close interdisciplinary collaboration to optimize outcomes for patients with rare or atypical manifestations of systemic vasculitis, while highlighting the need for further research to clarify the relationship between dupilumab and EGPA progression, as current literature does not clarify whether dupilumab triggers vasculitis or allows for the disease's progression, given no effect on treating the condition.
